# Molecular characterization of enterovirus-A71 in children with acute flaccid paralysis in the Philippines

**DOI:** 10.1186/s12879-019-3955-x

**Published:** 2019-05-02

**Authors:** Lea Necitas Apostol, Hiroyuki Shimizu, Akira Suzuki, Rifqiyah Nur Umami, Maria Melissa Ann Jiao, Amado Tandoc, Mariko Saito, Socorro Lupisan, Hitoshi Oshitani

**Affiliations:** 1 Department of Virology, Tohoku University Graduate School of Medicine, Sendai, Japan; 2 Department of Virology, Research Institute for Tropical Medicine, Muntinlupa, Philippines; 3 Department of Virology II, National Institute of Infectious Diseases, Musashimurayama, Tokyo Japan; 4 Tohoku–RITM Collaborating Research Center for Emerging and Re-emerging Infectious Diseases, Muntinlupa, Philippines; 5 Research Center for Biotechnology, Indonesian Institute of Sciences, Cibinong, 16911 Indonesia

**Keywords:** Acute flaccid paralysis, Enterovirus A71, Genogroup C2, Hand, Foot and Mouth disease, Philippines

## Abstract

**Background:**

Several inactivated enterovirus-A71 (EV-A71) vaccines are currently licensed in China; however, the development of additional EV-A71 vaccines is ongoing, necessitating extensive analysis of the molecular epidemiology of the virus worldwide. Until 2012, laboratory confirmation of EV-A71 for hand, foot, and mouth disease (HFMD) and other associated diseases had not occurred in the Philippines. Because EV-A71 has been linked with cases of acute flaccid paralysis (AFP), AFP surveillance is one strategy for documenting its possible circulation in the country. To expand current knowledge on EV-A71, molecular epidemiologic analysis and genetic characterization of EV-A71 isolates were performed in this study.

**Methods:**

A retrospective study was performed to identify and characterize nonpolio enteroviruses (NPEVs) associated with AFP in the Philippines, and nine samples were found to be EV-A71–positive. Following characterization of these EV-A71 isolates, the complete viral protein 1 (VP1) gene was targeted for phylogenetic analysis.

**Results:**

Nine EV-A71 isolates detected in 2000 (*n* = 2), 2002 (*n* = 4), 2005 (n = 2), and 2010 (*n* = 1) were characterized using molecular methods. Genomic regions spanning the complete VP1 region were amplified and sequenced using specific primers. Phylogenetic analysis of the full-length VP1 region identified all nine EV-A71 Philippine isolates as belonging to the genogroup C lineage, specifically the C2 cluster. The result indicated a genetic linkage with several strains isolated in Japan and Taiwan, suggesting that strains in the C2 cluster identified in the Asia-Pacific region were circulating in the Philippines.

**Conclusion:**

The study presents the genetic analysis of EV-A71 in the Philippines. Despite some limitations, the study provides additional genetic data on the circulating EV-A71 strains in the Asia-Pacific region, in which information on EV-A71 molecular epidemiology is incomplete. Considering that EV-A71 has a significant public health impact in the region, knowledge of its circulation in each country is important, especially for formulating vaccines covering a wide variety of strains.

## Background

Enterovirus-A71 (EV-A71) is an RNA virus belonging to the family *Picornaviridae*, genus *Enterovirus*, and species *EV-A*. Using the complete VP1 sequence for genetic classification, EV-A71 is divided into seven genogroups (A–G). Genogroup A contains only one prototype strain (BrCr), and genogroups B and C both have five subgenogroups (B1–B5 and C1–C5, respectively). D belongs to an Indian genogroup, E and F are of African origin, and G was found in India [1–5]. EV-A71 has caused outbreaks of hand, foot, and mouth disease (HFMD) and herpangina, and as a neurotropic virus, it can cause severe neurological complications such as aseptic meningitis, encephalitis, and poliomyelitis-like acute flaccid paralysis (AFP) in infants and young children with significant mortality and morbidity [2, 6–9]. Since its first isolation in 1969 in the USA, large outbreaks have been reported in different parts of the world, and the identification of EV-A71 infection has intensified since 1997, particularly in the Asia-Pacific region including Japan, Malaysia, Brunei, Taiwan, Australia, Singapore, China, and Vietnam [2, 10–12].

**Fig. 1:**
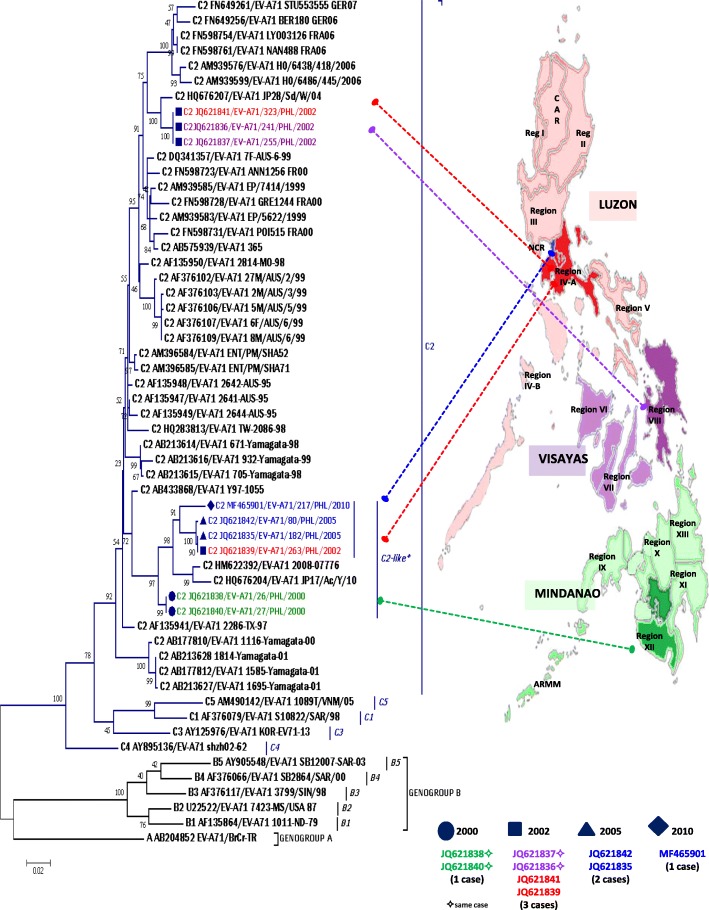
Phylogenetic tree of EV71 based on complete VP1 sequence and geographic location of EV71 isolates. The evolutionary history was inferred by using the Maximum Likelihood method based on the Tamura-Nei model. The percentage of trees in which the associated taxa clustered together is shown next to the branches. Initial tree(s) for the heuristic search were obtained automatically by applying Neighbor-Join and BioNJ algorithms to a matrix of pairwise distances estimated using the Maximum Composite Likelihood (MCL) approach, and then selecting the topology with superior log likelihood value. All positions containing gaps and missing data were eliminated. There were a total of 888 positions in the final dataset. Evolutionary analyses were conducted in MEGA7

Although EV-A71 is implicated in HFMD, which is usually a mild, self-limiting disease, the infection is also associated with severe neurologic diseases including aseptic meningitis, acute encephalitis, polio-like paralysis, acute brainstem encephalitis, cerebellar ataxia, and fulminant neurogenic pulmonary edema [13–15]. Much is known about the epidemiology of EV-A71 infection in many countries in the Asia-Pacific region, but little is known concerning its incidence, genetic diversity, and spectrum of disease in the Philippines. The Philippines is an archipelagic country with three geographical divisions that are further subdivided into 17 administrative regions (Luzon: Regions I, II, III, IV-A, IV-B, and V; Cordillera Administrative Region and National Capital Region [Visayas]: Regions VI, VII, and VIII; and Mindanao: Regions IX, X, XI, XII, XIII, and Autonomous Region for Muslim Mindanao, Fig. 1). Unlike neighboring countries, no specific surveillance for EV-A71 and HFMD was implemented in the Philippines. However, in line with the World Health Organization (WHO) Initiative on Global Poliomyelitis Eradication Program, AFP surveillance was implemented nationwide in 1992 to detect polioviruses and other enteroviruses. Based on the case definition by WHO, which is the detection of AFP in any child younger than 15 years, collection of two stool specimens within 14 days of the date of onset of paralysis is performed [16]. Since the establishment of AFP surveillance in the country, the national reference laboratory has detected large numbers of enteroviruses, including wild-type and vaccine-derived polioviruses, as well as other known enteroviruses. In the Philippines, outbreaks of HFMD and other related infections have been reported only via syndromic approaches, and laboratory identification of the causative agents including EV-A71 was not performed until July 2012. There has been no routine system in the Philippines for detecting circulating EV-A71 strains excluding those identified via AFP surveillance. Typing of nonpolio enteroviruses (NPEVs) identified during AFP surveillance in the Philippines, including eight EV-A71 strains, has been described [17]. With the significant impact of EV-A71 infection on human health and the need to develop an effective vaccine, defining the genetic characteristics of EV-A71 may expand current knowledge regarding its molecular epidemiology. Thus, this study focused on the detailed genetic characterization of EV-A71 strains associated with AFP to define the molecular characteristics of circulating strains in the Philippines.

## Methods

In total, 18,346 stool samples were collected from children presenting with AFP in the Philippines from 1992 to 2016, and 1332 NPEVs were isolated from these samples. Of these NPEVs, nine EV-A71 isolates were characterized in this study, including eight isolates identified as EV-A71 in a report published in 2012 using partial viral protein 1 (VP1) sequences [17] and an isolate detected in 2010. These isolates were tested using virus isolation and neutralization assays. Virus isolation was performed using a rhabdomyosarcoma cell line, and samples were identified as NPEV via the neutralization test using antisera pools provided by WHO, which contain antibodies against polioviruses and the most commonly observed enteroviruses [16]. All isolates that did not exhibit specific neutralization patterns were regarded as untypable NPEV. Because the antibody for EV-A71 was not included in the antisera pool, EV-A71 was not identified using the neutralization test. The serotypes of untypable NPEVs were further determined using a previously described molecular method for initial confirmation [17]. As the resulting amplicon size in the previous study was approximately 340 bp in size, the determination of genogroup identity may not be reliable. Therefore, amplification of the whole VP1 region, by which enteroviruses are classified, was performed. The complete VP1 genome (891 bp) was amplified using specific EV-A71 PCR primers, and the nucleotide sequence was determined. Nucleic acids were re-extracted from 0.1 ml of each infected tissue culture fluid using a High Pure viral nucleic acid kit (Roche Diagnostics, Mannheim, Germany) according to the manufacturer’s instructions. EV-A71–specific RT-PCR was performed using a single-step Access RT-PCR system (Promega, USA). The oligonucleotide primers employed for this assay were as follows: forward primer 71/2349, 5′-GCYTAYATAATAGCAYTGGCGGCAGC-3′; and reverse primer 71/3393, 5′-GGCGGTTRACCACYCTDAAGTTGCCCAC-3′. The RT-PCR assays were performed using a GeneAmp PCR system 2700 (Applied Biosystems, Foster City, CA, USA) with an uninterrupted thermocycling profile consisting of 45 min at 48 °C and 2 min at 94 °C to inactivate the AMV reverse transcriptase, followed by 35 cycles of 94 °C for 10 s, 50 °C for 10 s, and 65 °C for 1 min, and a final extension at 65 °C for 5 min. PCR products were visualized in 1% agarose gel with an expected product size of 1044 bp and purified using the Wizard SV Gel and PCR Clean-up system (Promega, USA) according to the manufacturer’s protocol.

The purified PCR products were labeled directly using a cycle sequencing reaction version 3.1 kit and analyzed using a 3100 Avant Genetic Analyzer (Applied Biosystems). Sequencher version 4.10.1 was used to assemble and generate consensus sequences determined from two strands and trimmed to obtain the full-length VP1 sequences using BioEdit v 4.7. MEGA 7.0 was then used to translate nucleotide sequences to amino acid sequences and construct phylogenetic trees using the maximum likelihood method with a bootstrap value of 1000 pseudoreplicates. The substitution model was employed using the Tamura-Nei method. The nucleotide sequences were deposited in GenBank under the following accession numbers: JQ621835 (C2/EV-A71/182/PHL/2005), JQ621836 (C2/EV-A71/241/PHL/2002), JQ621837 (C2/EV-A71/255/PHL/2002), JQ621838 (C2/EV-A71/26/PHL/2000), JQ621839 (C2/EV-A71/263/PHL/2002), JQ621840 (C2/EV-A71/27/PHL/2000), JQ621841 (C2/EV-A71/323/PHL/2002), JQ621842 (C2/EV-A71/80/PHL/2005), and MF465901(C2/EV-A71/217/PHL/2010).

## Results

EV-A71 was detected in 9 of 1332 NPEV-positive stool samples (0.70%) collected between 1992 and 2016 from seven children with AFP (Table 1). Of these seven AFP cases, two children had stools positive for EV-A71 in Regions XII (26/PHL/2000 and 27//PHL/2000) and VIII (241/PHL/2002 and 255/PHL/2002). Phylogenetic analysis of the full-length VP1 region illustrated that all nine Philippine EV-A71 isolates belonged to genogroup C, especially the C2 and C2-like clusters (Fig. 1). The Philippine EV-A71 strains (80/PHL/2005 [JQ621842]; 182/PHL/2005 [JQ621835]; 263/PHL/2002 [JQ621839]; 217/PHL/2010 [MF465901]; 26/PHL/2000 [JQ621838], and 27/PHL-2000 [JQ621840]) were closely related to a previously reported C2-like subgenogroup [18], especially a Taiwanese isolate obtained in 2008 (GenBank: HM622392) and a Japanese strain detected in 2010 (GenBank: HQ676204) with divergence ranges of 0.028–0.031 and 0.031–0.037 nucleotides, respectively. Interestingly, the Philippine isolates of the C2-like clade formed two independent subclusters, including one cluster for isolates 80/PHL/2005, 182/PHL/2005, 263/PHL/2002, and 217/PHL/2010 and another cluster for 26/PHL/2000 and 27/PHL/2000. The other three EV-A71 Philippine isolates (241/PHL/2002, 255/PHL/2002, and 323/PHL/2002) were genetically similar to a Japanese strain isolated in 2004 (GenBank: HQ676207) with 0.013-nucleotide divergence (Table 2). The high homology of these isolates suggests that similar C2 strains are widely circulating in the Asia-Pacific region. Another significant finding was the isolation of 323/PHL/2002 in Region IV-A, exhibiting perfect homology with strains isolated in Region VIII from one patient (255/PHL/2002 and 241/PHL/2002) in the same year. A similar finding was also observed for 263/PHL/2002 isolated in 2002, which had high homology with 80/PHL/2005 and 182/PHL/2005 isolated in 2005 in different geographical areas. All Philippine strains had homologous strains in other Asian countries.

**Table 1:** Distribution of NPEV and EV-A71 isolated and detected per year, Acute Flaccid Paralysis Surveillance, Philippines, 1992–2016

Year of Isolation	Number of NPEV Isolate	Number of EV-A71 Isolate	Number of EV-A71 Case	EV-A71 Positive Rate (%)
1992	16	0	–	–
1993	3	0	–	–
1994	10	0	–	–
1994	11	0	–	–
1996	43	0	–	–
1997	115	0	–	–
1998	64	0	–	–
1999	27	0	–	–
2000	77	2	1	2.6
2001	69	0	–	–
2002	93	4	3	4.3
2003	58	0	–	–
2004	26	0	–	–
2005	37	2	2	5.4
2006	44	0	–	–
2007	51	0	–	–
2008	54	0	–	–
2009	82	0	–	–
2010	50	1	1	2.0
2011	63	0	–	–
2012	77	0	–	–
2013	72	0	–	–
2014	21	0	–	–
2015	100	0	–	–
2016	69	0	–	–
TOTAL	1332	9	7	0.7

**Table 2:** Estimates of Evolutionary Divergence of subgenogroup C2 with EV-A71 Philippine isolates

Case (Location)	EV-A71 strain	JQ621838 JQ621840	JQ621836 JQ621837	JQ621841	JQ621839	JQ621842	JQ621835	MF465901	HM 622392	HQ 676207	HQ 676204	AB 433868
1 (Region XII)	JQ621838 (26/PHL/2000) JQ621840 (27/PHL/2000)	X										
2 (Region VIII)	JQ621836 (241/PHL/2002) JQ621837 (255/PHL/2002)	0.044	X									
3 (Region IV-A)	JQ621841 (323/PHL/2002)	0.044	0	X								
4 (Region IV-A)	JQ621839 (263/PHL/2002)	0.026	0.054	0.054	X							
5 (Metro Manila)	JQ621842 (80/PHL/2005)	0.026	0.054	0.054	0.001	X						
6 (Metro Manila)	JQ621835 (182/PHL/2005)	0.026	0.054	0.054	0.000	0.001	X					
7 (Metro Manila)	MF465901 (S10–217/ PHL/2010)	0.030	0.059	0.024	0.024	0.024	0.024	X				
Reference Strain Taiwan	HM622392 (2008–07776)	0.028	0.056	0.056	0.028	0.028	0.028	0.031	X			
Reference Strain Japan	HQ676207 (JP28/Sd/W/04)	0.044	0.013	0.013	0.054	0.054	0.054	0.059	0.058	X		
Reference Strain Japan	HQ676204 (JP17/Ac/Y/10)	0.032	0.056	0.056	0.031	0.031	0.031	0.037	0.013	0.060	X	
Reference Strain Japan	AB433868 (Y97–1055)	0.022	0.032	0.032	0.038	0.038	0.038	0.042	0.040	0.032	0.043	X

X indicates value not calculated because the diagonal values would be homologous comparisons. Data represent number of base substitutions per site from between sequences. Standard error estimate(s) are shown above the diagonal and were obtained by a bootstrap procedure (1000 replicates). Analyses were conducted using the Maximum Composite Likelihood model. All positions containing gaps and missing data were eliminated. Evolutionary analyses were conducted in MEGA7

## Discussion

This is the first report of the genetic analysis of EV-A71 strains in the Philippines. The result of previous studies suggested that EV-A71 genogroup B predominates in Southeast Asia and that genogroup C prevails in East Asian countries such as Japan, Taiwan, Korea, and China [2, 19]. EV-A71 outbreaks have been frequently reported in Taiwan, Vietnam, and Malaysia, and considering the geographic location of the Philippines and the highly contagious tendency of EV-A71, it can be assumed that EV-A71 also circulated in the Philippines during the same periods of circulation in neighboring countries.

EV-A71 genogroup C was detected for the first time in the late 1980s (C1–1986-Australia; C2–1995-Australia; C3–2000-Korea; C4–1998-Taiwan and; C5–2005-Vietnam) [20], and it has since been circulating in the Western Pacific region, the USA, and European countries such as Norway, France, Germany, Austria, the United Kingdom, and the Netherlands, indicating that genogroup C circulates globally [21–26]. However, the geographic circulation of subgenogroups within genogroup C appears to be restricted or limited because subgenogroups C3, C4, and C5 have only been observed in Asian countries. The present report, which used a longitudinal molecular characterization to analyze the entire VP1 region of EV-A71 implicated in AFP in the Philippines, further supports the continuous circulation of genogroup C in Asian countries including the Philippines.

Based on the available complete VP1 sequences detected between 1970 and 2008, genogroup C is more prevalent than genogroup B; however, the pattern of outbreaks in several Asian countries such as Taiwan indicated the alternating predominance of genogroups C2, B4, C4, B5, and C5 [20]. In the present study, only C2 and C2-like subgenogroups were detected in the Philippines between 2000 and 2010. Among the genogroup C strains detected in the Asia-Pacific region, C2 is apparently not a dominant subgenogroup, as its occurrence is extremely low outside Japan, in which constant and uninterrupted circulation of C2 was observed from 1997 to 2002 [27]. The circulation of C2 was further observed following HFMD outbreaks in Malaysia, Taiwan, Australia, Thailand, and Singapore between 1997 and 2008 [2, 28–31]. Although global herd immunity against C1 and C2 may explain the possible prevalence of epidemics caused by subgenogroups B4 and C4 in the Asia-Pacific region, previous studies revealed the recurrence of C2 [21, 23, 32]. The last reported detection of C2 in the Asia-Pacific region was reported in Japan in 2013. Although C2 was not detected in the region between 2003 and 2005, the emergence of C2 was documented in Thailand and South Korea in 2006 and 2009, respectively. Likewise, subgenogroup C2 reappeared in Japan in 2007–2010 [31, 33]. In 2010, a study from Taiwan reported EV-A71 strains (HM622392 and HM622391) belonging to C2-like subgenogroup [18]. The finding of these two C2-like strains collected from different patients in different month in 2008 indicates the circulation of the C2-like viruses at a certain period of time in Taiwan. The phylogenetic analysis in this Taiwan study based on the P1 and P2 regions of the C2-like strains forming a genetically distinct cluster revealed a possibility of designating a new subgenogroup. Based on the complete VP1 sequence, six Philippine EV-A71 isolates (JQ621835, JQ621838, JQ621839, JQ621840, JQ621842 and MF465901) clearly belonged to the subgenogroup C2 and further clustered with the reported Taiwan C2-like strains. To know whether the Philippine EV-A71 isolates will form a separate genetic cluster similar to the Taiwan C2-like strains, a full-length genome analysis is needed. Nonetheless, the isolation of C2-like strains in the Philippines prior to its first detection in Taiwan in 2008 gives evidence to the existence of a C2-like subgenogroup and suggests that the Philippine EV-A71 C2-like strains may be the ancestors of the Taiwan C2-like isolates.

During this 25-year period, we found only EV-A71 genogroup C, in particular, subgenogroup C2 in 2002 and subgenogroup C2-like in 2000, 2002, 2005, and 2010 in different geographic locations in the Philippines (Fig. 1). Although the samples characterized in this study were obtained from patients with AFP, the finding of a monophyletic C2 does not dismiss the fact that it may have been the most prevalent genogroup in 2000–2010, if not the sole subgenogroup present in the country. Whether co-circulation with other EV-A71 genogroups exists in the Philippines remains unknown, and further monitoring of other EV-A71–related diseases, particularly HFMD, is required. Central nervous system involvement as a clinical symptom of AFP is extremely rare. Therefore, the current molecular characterization of nine EV-A71 isolates from patients with AFP may not reveal the entire picture of circulating strains in the country.

Generally, enteroviruses use two evolutionary mechanisms, namely mutation and recombination. Studies have demonstrated the presence of both intra- and intertypic EV-A71 recombinants [34–37]. In both phenomena, altered virologic characteristics have been noted such as changes in antigenicity [18]. Based on two published studies of C2 recombination events, no C2 recombinant was found in the Netherlands; however, two strains from Taiwan (HM622391 and HM622392) clustered as C2-like strains exhibited intratypic recombination [18, 38, 39]. When the Philippine EV-A71 isolates were compared with other strains belonging to the same subgenogroup, the most homologous strain related to the six Philippine strains (26/PHL/2000, 27/PHL/2000, 80/PHL/2005, 182/PHL/2005, 263/PHL/2002, 217/PHL/2010) was the C2-like Taiwanese strain HM622392. HM622392 is a recombinant virus that displayed a significant difference in the neutralization test, having up to a 128-fold lower neutralization antibody titer than other C2 strains [18]. Whether the Philippine EV-A71 isolates share phenotypic and antigenic characteristics with the known Taiwan strain with a low cross-neutralizing titer against existing C2 is unknown, and this warrants a full sequence analysis and determination of neutralization antibody titers. Another finding based on the sequence analysis in this study was the identification of EV-A71 strains with perfect homology in the VP1 region isolated in the same year, namely 255/PHL/2002 (JQ621837) and 241/PHL/2002 (JQ621836) from Region VIII, and 323/PHL/2002 (JQ621841) from Region IV-A. Further investigation of these cases revealed no travel history among the patients, suggesting that certain strains were co-circulating in 2002 in the entire country.

Although the present study only examined samples from patients with AFP opposed to a diverse group of individuals presenting with other clinical conditions, this study on EV-A71–associated AFP provides a focal premise that Philippine EV-A71 strains may have neurovirulent potential similarly as strains found in the Asia-Pacific region. Given the partial extent of EV-A71 detection in this study, a significant observation is that subgenogroup C2 might have a role in a broad spectrum of diseases. Many of the subgenogroup C2 strains detected previously were associated with fatal HFMD, aseptic meningitis, myelitis, encephalitis, bronchiolitis, and herpangina, and samples in this study were comprehensively obtained via 25 years of virologic surveillance of AFP-associated illness.

## Conclusions

This study reports the existence of only EV-A71 C2 subgenogroup in the Philippines, indicating that this subgenogroup is indigenously circulating in the country because it was isolated from different geographic areas. Although large outbreaks of EV-A71 infection have been described in the Asia-Pacific region, no outbreak has occurred in the Philippines. The present work could be used for future genetic detection studies on EV-A71 on a much larger scale to determine the overall epidemiology in the country. Given the potential health threat of the virus, EV-A71 should be monitored in the Philippines. Although limited in scope, the present study provides further information on the genetic pool of circulating EV-A71 strains in the Asia-Pacific region.

## Abbreviations

AFP: acute flaccid paralysis
EV-A71: Enterovirus 71
HFMD: hand, foot and mouth disease
NPEV: nonpolio enterovirus
PCR: polymerase chain reaction
VP1: viral protein 1
WHO: World Health Organization
